# IgG4-Related Disease Is Not Associated with Antibody to the Phospholipase A2 Receptor

**DOI:** 10.1155/2012/139409

**Published:** 2012-05-10

**Authors:** Arezou Khosroshahi, Rivka Ayalon, Laurence H. Beck, David J. Salant, Donald B. Bloch, John H. Stone

**Affiliations:** ^1^Department of Medicine, Harvard Medical School and Rheumatology Unit, Massachusetts General Hospital, Boston, MA 02114, USA; ^2^Section of Nephrology, Department of Medicine, Boston University School of Medicine, Boston, MA 02118, USA; ^3^Department of Medicine, Harvard Medical School and The Division of Rheumatology, Allergy and Immunology and The Center for Immunology and Inflammatory Diseases of the General Medical Services, Massachusetts General Hospital, Boston, MA 02114, USA

## Abstract

Patients with IgG4-related disease (IgG4-RD) share histopathological characteristics that are similar across affected organs. The finding of infiltration with IgG4+ plasma cells in the proper clinical and histopathological contexts connects a large number of clinical entities that were viewed previously as separate conditions. The renal involvement in IgG4-RD is usually characterized by tubulointerstitial nephritis, but membranous nephropathy has also been reported to be one of the renal complications of IgG4-RD. The recent discovery that a high proportion of patients with idiopathic membranous nephropathy (IMN) have IgG4 autoantibodies to the M-type phospholipase A2 receptor (PLA2R) in the circulation and glomerular immune deposits, together with the profound IgG4 hypergammaglobulinemia and occasional reports of membranous nephropathy in IgG4-RD, raised the question of a common antigen. To assess the presence of anti-PLA2R antibody in patients with IgG4-RD, we screened sera from 28 IgG4-RD patients by immunoblot. None of the patients in this cohort had detectable circulating anti-PLA2R antibodies. This study suggests that despite some clinical and serological overlaps between IgG4-RD and IMN,anti-PLA2R antibodies do not play a role in the pathogenesis of IgG4-RD. Additional studies of IgG4-RD with evidence of membranous nephropathy are important to exclude any definite relationship.

## 1. Introduction

IgG4-related disease (IgG4-RD) is a multiorgan system fibroinflammatory condition defined by a tendency to form tumorous lesions in various organs including the pancreas, salivary and lacrimal glands, biliary tract, liver, lung, and kidney, aorta [1]. The histopathologic findings are remarkably similar across all organs in this disease. The distinctive pathologic features include a dense lymphoplasmacytic infiltrate rich in IgG4-positive plasma cells, storiform fibrosis, obliterative phlebitis, and eosinophilia [2]. Frequent elevations of serum IgG4 in patients with IgG4-RD and significant clinical responses to glucocorticoids are other hallmarks of this condition [3]. The relationship between elevated serum IgG4 and distinctive patterns of organ involvement was first recognized in autoimmune pancreatitis [4], but subsequent observations led to the identification of this disease in nearly all organ systems [1, 2, 5].

Idiopathic membranous nephropathy (IMN) is an organ-specific autoimmune disorder and a leading cause of nephrotic syndrome in adults. Until recently, the etiology of this condition was unknown, but studies in experimental MN had established that circulating antibodies bind to a target antigen on glomerular podocytes and form antigen-antibody complexes that cause podocyte injury and proteinuria [6]. In 2009, Beck et al. discovered that a high proportion of patients with IMN have circulating IgG4 autoantibodies that bind to the M-type phospholipase A2 receptor (PLA2R), a transmembrane glycoprotein, and member of the mannose receptor family expressed on human glomerular podocytes [7]. This finding is congruent with previous reports that IgG4 predominates in the immune deposits of renal biopsy specimens of IMN. This predominance of IgG4 is not observed in secondary—or lupus-associated—membranous nephropathy [8]. Studies of patients in several different cohorts have indicated that 70–80% of patients with IMN have anti-PLA2R antibodies that are of the IgG4 subclass [7, 9–11]. Of note, however, hypergammaglobulinemia and elevated serumIgG4 concentrations are not reported in IMN patients.

IgG4-RD and IMN both appear to respond well to B cell depletion treatment with rituximab [9, 12, 13]. The early experience with B cell depletion in IgG4-RD suggests that rituximab (RTX) has a targeted effect on serum IgG4 : IgG4 decreases rapidly following B cell depletion while the concentrations of other IgG subclasses remain stable [12, 13]. RTX has also been reported in case series to be effective in IMN [14, 15]. A decline in anti-PLA2R antibodies has been shown to precede the clinical improvement of patients with membranous nephropathy [9]. A randomized clinical trial of RTX in IMN is now under way (Clinicaltrials.gov identifier NCT01180036).

Membranous nephropathy has been reported in some patients with IgG4-RD [16–18], but the principal renal manifestation of IgG4-RD is tubulointerstitial nephritis [19, 20], characterized by interstitial fibrosis and infiltration of lymphocytes and IgG4-positive plasma cells. Immune complex deposition and membranous glomerulonephritis have been shown to coexist with tubulointerstitial nephritis in a minority of patients with IgG4-RD [21, 22]. Cravedi et al. [23] recently described a patient with IgG4-RD who had pancreatic and salivary gland involvement and subsequently developed proteinuria. A renal biopsy showed features of membranous nephropathy. A search for anti-PLA2R antibodies in that patient's serum was negative. Likewise, anti-PLA2R antibodies were not detected in the case of IgG4-RD and membranous nephropathy reported by Fervenza et al. [18]. 

Because of certainclinical and pathological features of IgG4-RD and IMN overlap, the shared association with antibodies of the IgG4 subclass, and the ostensible improvement that both diseases demonstrate in response to B cell depletion, we assayed sera from patients in our longitudinal IgG4-RD registry for antibodies directed against PLA2R.

## 2. Material and Methods

### 2.1. Patients

Between July 2009 and September 2011, we obtained serum samples from 28 patients with IgG4-RD. All patients were enrolled in the Massachusetts General Hospital IgG4-RD Registry. The screening of human sera for anti-PLA2R antibodies was approved by the Institutional Review Boards at both the Massachusetts General Hospital and Boston University Medical Center.

### 2.2. Inclusion Criteria for the IgG4-RD Registry

Patients were eligible to participate in the study if they had a biopsy-confirmed diagnosis of IgG4-RD. Histopathologic features considered to be highly suggestive of IgG4-RD diagnosis included lymphoplasmacytic infiltrates and storiform fibrosis within involved organs. Obliterative phlebitis and mild-to-moderate tissue eosinophilia were observed frequently but were not required for the diagnosis [24]. In addition, all patients had either an IgG4/IgG plasma cell ratio of >50% within the affected organs or more than 30 IgG4-bearing plasma cells/high-power field (hpf). Elevated serum IgG4 was not required for the diagnosis of IgG4-RD.

### 2.3. IgG4 Plasma Cell Quantitation

Immunohistochemical staining was performed as previously described [12]. Formalin-fixed, paraffin-embedded tissue sections were stained with antibodies to IgG4 (Zymed, 1 : 200 dilution) or IgG (Dako, 1 : 3000 dilution). For each specimen, the number of IgG4+ plasma cells and the total number of IgG+ plasma cells were assessed in three nonoverlapping high-power fields (400x). The three fields with the highest number of IgG4+ plasma cells were selected for quantitation, and the ratio of IgG4+/IgG+ plasma cells was determined.

### 2.4. Serum IgG4 Assay

Serum IgG4 concentrations were measured by nephelometry (Mayo Medical Laboratories New England, Andover, Massachusetts).

### 2.5. Immunoblot Assay for the Detection of Anti-PLA2R Antibodies

Patients' sera were tested for the presence of anti-PLA2R antibodies by immunoblot, under nonreducing conditions, as previously described [7]. Recombinant human PLA2R was fractionated by polyacrylamide gel electrophoresis and transferred to nitrocellulose membranes. Individual lanes were cut and incubated overnight at 4°C with serum samples diluted 1 : 25 in Tris-buffered saline containing 0.2% Tween-20 and 10% skim milk. Serum from a patient with IMN, previously shown to have anti-PLA2R antibodies, was used as a positive control. IgG subclass-specific sheep anti-human IgG4 (The Binding Site, San Diego, CA) was used at 1 : 3000. Sheep IgG was subsequently detected with species-specific, horseradish peroxidase-conjugated donkey anti-sheep IgG (Jackson ImmunoResearch, West Grove, PA), followed by reaction in a chemiluminescent substrate and exposure to radiographic film for two minutes. A band corresponding to the size of PLA2R was judged to represent the presence of anti-PLA2R antibodies.

## 3. Results

**Demographic Features**

Sera from 28 patients were tested for the presence of anti-PLA2R antibodies. The patients' baseline characteristics are shown in Table 1.The IgG4-RD patients included 15 men and 13 women, with an average age of 57 years (range: 24–82).

**Clinical manifestations of IgG4-RD**

The patients' manifestations of IgG4-RD covered the full range of disease expression [25], including clinically evident renal disease in two patients (see below). Multiorgan system IgG4-RD was observed in 11 patients (39%). The most commonly involved organs and tissues were the lymph nodes (*n* = 9), salivary glands (*n* = 8), and orbital regions (*n* = 7). Five patients had IgG4-related pancreatitis (type 1 AIP), and four had IgG4-related sclerosing cholangitis. The other sites involved were the retroperitoneum (*n* = 4), aorta (*n* = 3), and the skin, pericardium, lung, thyroid gland, and tonsils (1 patient each).

**Renal Disease**

Two patients had evidence of kidney dysfunction, characterized by renal masses and proteinuria. Patient 16 [26] was found to have bilateral kidney masses on a magnetic resonance imaging study performed for evaluation of IgG4-related sclerosing cholangitis. Renal biopsy showed a destructive tubulointerstitial infiltrate with prominent fibrosis. The inflammatory infiltrate was composed predominantly of IgG4+ plasma cells. There were significant quantities of electron dense deposits within the thickened tubular basement membrane, and small electron-dense deposits were seen scattered within the fibrotic interstitium. The glomeruli were free of deposits.

Patient 14 was diagnosed with IgG4-related retroperitoneal fibrosis. Acute renal failure and proteinuria emerged a year after the diagnosis of IgG4-RD and resolved with glucocorticoid treatment. Kidney biopsy showed tubular injury, periglomerular fibrosis, interstitial inflammation, and the presence of IgG4+ plasma cells. Immunofluorescence microscopy showed no tubular basement membrane or glomerular basement membrane deposits. There were prominent mesangial granular deposits of IgG, IgA, IgM, and C3.

**Treatment before Serum Sampling**

Most of the patients were entered into the IgG4-RD Registry after the initiation of prednisone treatment. Twenty-one (75%) of the 28 patients were receiving glucocorticoids at the time their serum IgG4 was measured for the first time. Fifteen (54%) of the 28 patients had elevated serum IgG4. Among those patients with elevated serum IgG4 concentrations at baseline, the mean IgG4 was 640 mg/dL (normal <135 mg/dL). The mean serum IgG4 concentration for all patients in the cohort was 342 mg/dL. Among the seven patients who were not treated with glucocorticoids before the diagnosis of IgG4-RD and serum sampling, the mean IgG4 level was 230 mg/dL.

**Other Autoantibody Testing**

Patients were tested for rheumatoid factor by nephelometry, which was positive in 2 patients (7%). Antinuclear antibody (ANA) testing by immunofluorescence using the HEp-2 substrate was positive in 24 patients (85%). Patient 4 had a strongly positive ANA in an anticentromere pattern but had no clinical features of limited systemic sclerosis or Sjögren's syndrome, and salivary gland histopathology disclosed findings diagnostic of IgG4-RD, not Sjögren's syndrome. Enzyme-linked immunoabsorbent assays for antibodies against Ro, La, Sm, and RNP antigens were negative in all 28 patients.

**Anti-PLA2R antibody assays**

**Figure 1:**
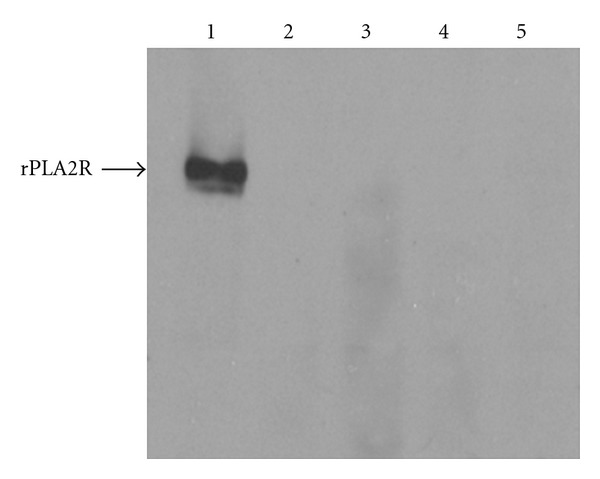
Representative immunoblot demonstrating that sera from patients with IgG4-RD (lanes 2–5) lack detectable reactivity with rPLA2R. Serum from a patient with idiopathic membranous nephropathy (lane 1) was used as a positive control.

The sera from these 28 patients with IgG4-RD were tested for the presence of autoantibodies reactive with PLA2R. None of the serum samples contained detectable anti-PLA2R antibodies when screened at a dilution of 1 : 25 (Figure 1).

## 4. Discussion

IgG4-RD and IMN have significant overlap in their renal manifestations. Although membranous nephropathy is most often a renal limited autoimmune disease (so-called idiopathic or primary MN), membranous lesions and nephrotic syndrome have also been reported in IgG4-RD [23] and may accompany the more usual interstitial nephritis typical of IgG4-RD [16, 27]. Both IgG-RD and IMN are associated with perturbations in the IgG4 antibody subclass. The autoantibody recently linked to IMN is generally of the IgG4 subclass, and IgG4 hypergammaglobulinemia—occasionally present up to 25 times the upper limit of normal—occurs in 70% of patients with IgG4-RD [28]. In addition, many cases of both IMN and IgG4-RD are exquisitely sensitive to treatment with rituximab. B cell depletion is associated with declines in the titers of anti-PLA2R in IMN and the level of IgG4 hypergammaglobulinemia in IgG-RD. Despite the similarities between IMN and IgG4-RD, the principal finding of this study is that the likelihood of a relationship between IgG4-RD and autoantibodies to the PLA2R is low. These antibodies were not identified in any of the 28 patients evaluated in this study. The absence of anti-PLA2R in two previous cases of IgG4-RD accompanied by membranous nephropathy is noteworthy [18, 23].

There are a variety of potential explanations for this finding. The first is that anti-PLA2R antibodies do not play a role in IgG4-RD and that a different antigen-antibody system is at play in those few cases that develop membranous nephropathy [23]. This is consistent with the fact that although important similarities exist between these two conditions, fundamental differences also exist. Whereas IMN is a renal-limited lesion, IgG4-RD is a multiorgan disease [25]. IMN patients do not demonstrate elevated serum IgG4 and decreased complement levels which are seen in some patients with IgG4-RD. Furthermore, although membranous GN has been described in IgG4-RD, the renal lesion most characteristic of IgG4-RD is tubulointerstitial nephritis. Finally, the other pathological features that are central to IgG4-RD, namely, storiform fibrosis, obliterative phlebitis, and the infiltration of large numbers of IgG4+ plasma cells are absent in IMN. The glomerular lesions of IgG4-RD described to date have included mesangial proliferative glomerulonephritis, membranous nephropathy, membranoproliferative glomerulonephritis, and endocapillary proliferative glomerulonephritis [19, 29].

A second possible explanation for our inability to detect the anti-PLA2R antibodies in this IgG4-RD patient cohort is that 75% of the patients received glucocorticoids prior to their entry to the study. Although glucocorticoids may have reduced the level of anti-PLA2R antibodies below the level of detection by the immunoblot assay used in this study, they are generally ineffective when used alone for treatment of IMN. Moreover, we did not detect these antibodies even in patients who had not been treated with glucocorticoids prior to serum sampling (*n* = 7).

A third potential explanation for our failure to find any relationship between the presence of anti-PLA2R antibodies and IgG4-RD is that IgG4-RD is not a single disease but rather a pathologic syndrome in which certain mechanisms operate across organ systems. If this explanation were true, then anti-PLA2R antibodies might play a role in some disease subsets of IgG4-RD, particularly the renal disease subset. The renal disease subset of IgG4-RD is underrepresented in our Registry as only two patients had overt renal disease, and none had biopsy-proven membranous glomerulonephropathy or nephrotic range proteinuria. An expanded study that includes a larger number of IgG4-RD patients with renal involvement of this nature is important to exclude definitively any relationship between IgG4-RD and antibodies to the PLA2R.

Given the consistency of pathological features across involved organs in IgG4-RD, we believe that our findings likely represent the true nature of the relationship between IgG4-RD and antibodies to the PLA2-R, namely, that there is none. The significance of IgG4 hypergammaglobulinemia and IgG4+ plasma cell infiltration into involved organs in patients with IgG4-RD—whether they are pathogenic or just “innocent bystanders”—remains to be clarified.

## Figures and Tables

**Table 1:** IgG4-RD patients' characteristics.

Patient	Age	Sex	Serum IgG4 mg/dL	Serum IgG mg/dL	ANA	Prednisonetreatment	Organ involved
1	67	M	1340	2450	+1 : 1280 S	Yes	aorta, lymph node
2	76	M	2050	2310	+1 : 40 S	Yes	pancreas, biliary tract, aorta, lymph node, salivary gland
3	55	M	1500	1640	+1 : 160 S	Yes	lacrimal and salivary glands
4	51	F	401	726	+1 : 5120 C	Yes	lacrimal and salivary glands
5	60	M	365	2570	+1 : 40 S	Yes	aorta
6	58	M	894	1210	+1 : 40 S	No	lymph node
7	41	M	200	766	+1 : 40 H	No	lymph node
8	24	M	47	100	—	Yes	orbital tissue
9	72	F	429	1170	+1 : 320 S	Yes	lacrimal & salivary glands Skin
10	52	F	62	1280	—	Yes	biliary tract
11	40	F	98	1090	+1 : 160 S	Yes	pancreas, lung, thyroid, lacrimal gland
12	59	M	27	1280	+1 : 40 S	Yes	retroperitoneum
13	48	F	9	1240	+1 : 40 S	No	pericardium
14	63	M	386	1500	+1 : 40 S	Yes	retroperitoneum, kidney
15	56	M	670	1980	+1 : 640 H	Yes	orbital tissue
16	74	F	28	1830	+1 : 40 S	Yes	kidney, lacrimal gland, pancreas, and biliary tract
17	39	M	53	1460	+1 : 40 S	Yes	retroperitoneum
18	51	F	68	1190	+1 : 40 S	No	lymph node
19	33	F	139	1570	+1 : 160 S	No	lymph node
20	58	F	194	1160	+1 : 40 H	Yes	tonsils
21	41	F	240	1710	+1 : 160 H	No	salivary gland
22	63	F	30	923	+1 : 320 S	Yes	orbital tissue
23	51	F	80	1300	—	Yes	pachymeninges
24	82	M	59	1180	—	No	lymph node
25	66	F	154	769	+1:160 S	Yes	retroperitoneum, lymph node, salivary gland
26	72	M	12	1000	+1 : 40 S	Yes	pancreas, salivary gland
27	70	M	164	1230	+1 : 160 S	Yes	pancreas, biliary tract, salivary gland
28	61	M	50	1650	+1 : 160 S	Yes	pancreas, retroperitoneum, hard palate, lymph node

S: Speckled H: Homogeneous C: Centromere.
